# Supplementary material for: An in vitro platform for the enzymatic characterization of the rhomboid protease RHBDL4
Source: bioRxiv. 2024 Oct 13:2024.10.13.618094. Preprint. [Version 1] doi: 10.1101/2024.10.13.618094 (PMC11483055; doi:10.1101/2024.10.13.618094)
Supplement: Supplementary file 1 [file NIHPP2024.10.13.618094v1-supplement-1.pdf]

bioRxiv preprint doi: https://doi.org/10.1101/2024.10.13.618094; this version posted October 13, 2024. The copyright holder for this preprint (which was not certified by peer review) is the author/funder, who has granted bioRxiv a license to display the preprint in perpetuity. It is made available under aCC-BY 4.0 International license.

Supplemental Figures

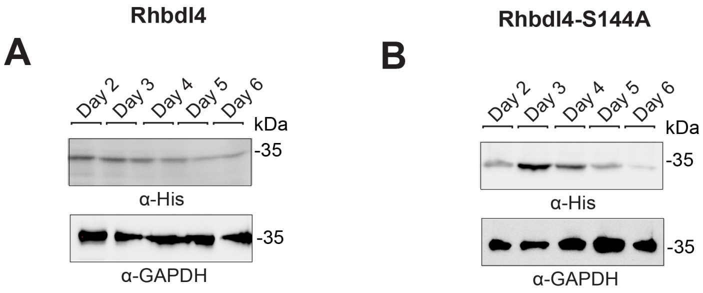

Supplemental Figure 1. (A) Freestyle 293-F suspension cells were transfected with the RHBDL4 plasmid and cells were grown for the indicated number of days. 50 µg of lysate was subjected to immunoblotting for polyclonal α-RHBDL4 antibody and monoclonal α-GAPDH antibody. **(B)** Same as (A) except RHBDL4-S144A was transfected.

bioRxiv preprint doi: https://doi.org/10.1101/2024.10.13.618094; this version posted October 13, 2024. The copyright holder for this preprint (which was not certified by peer review) is the author/funder, who has granted bioRxiv a license to display the preprint in perpetuity. It is made available under aCC-BY 4.0 International license.

A

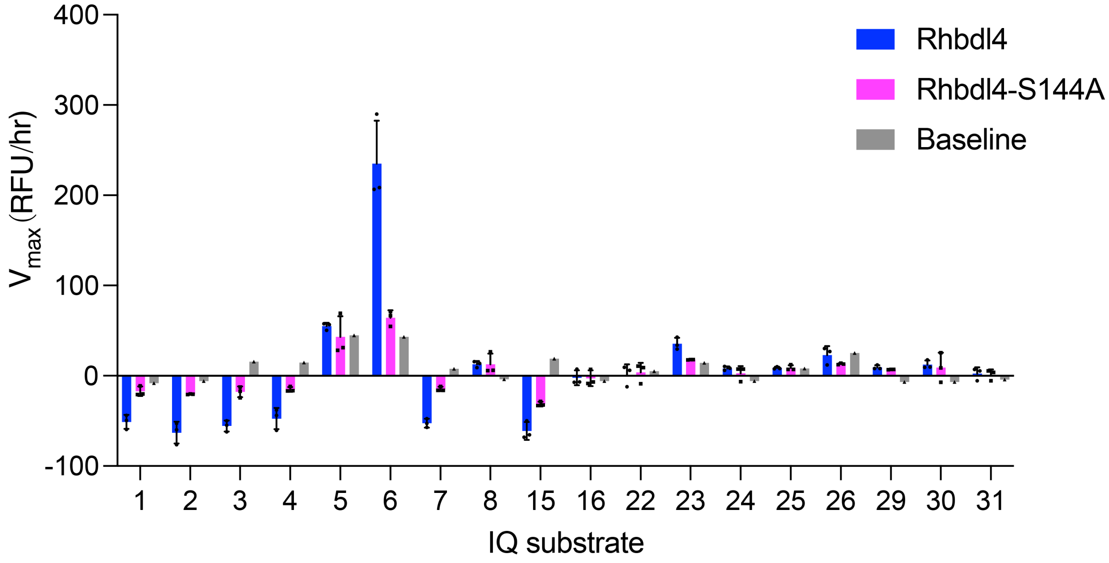

B

IQ6	
K_M(μM)	10.73±2
K_{cat}(hr^{-1})	7.33±0.6
K_{cat}/K_M(μM^{-1}hr^{-1})	.68±0.04

IQ41	
K_M(μM)	5.63±2
K_{cat}(hr^{-1})	2.41±0.6
K_{cat}/K_M(μM^{-1}hr^{-1})	.43±0.02

IQ46	
K_M(μM)	7.19±1
K_{cat}(hr^{-1})	3.62±0.8
K_{cat}/K_M(μM^{-1}hr^{-1})	.50±0.05

Supplemental Figure 2. (A) Proteolytic rate of indicated IQ substrates cleaved by RHBDL4. **(B)** Catalytic parameters of IQ6, IQ40, and IQ41 substrate cleavage by RHBDL4.

bioRxiv preprint doi: https://doi.org/10.1101/2024.10.13.618094; this version posted October 13, 2024. The copyright holder for this preprint (which was not certified by peer review) is the author/funder, who has granted bioRxiv a license to display the preprint in perpetuity. It is made available under aCC-BY 4.0 International license.

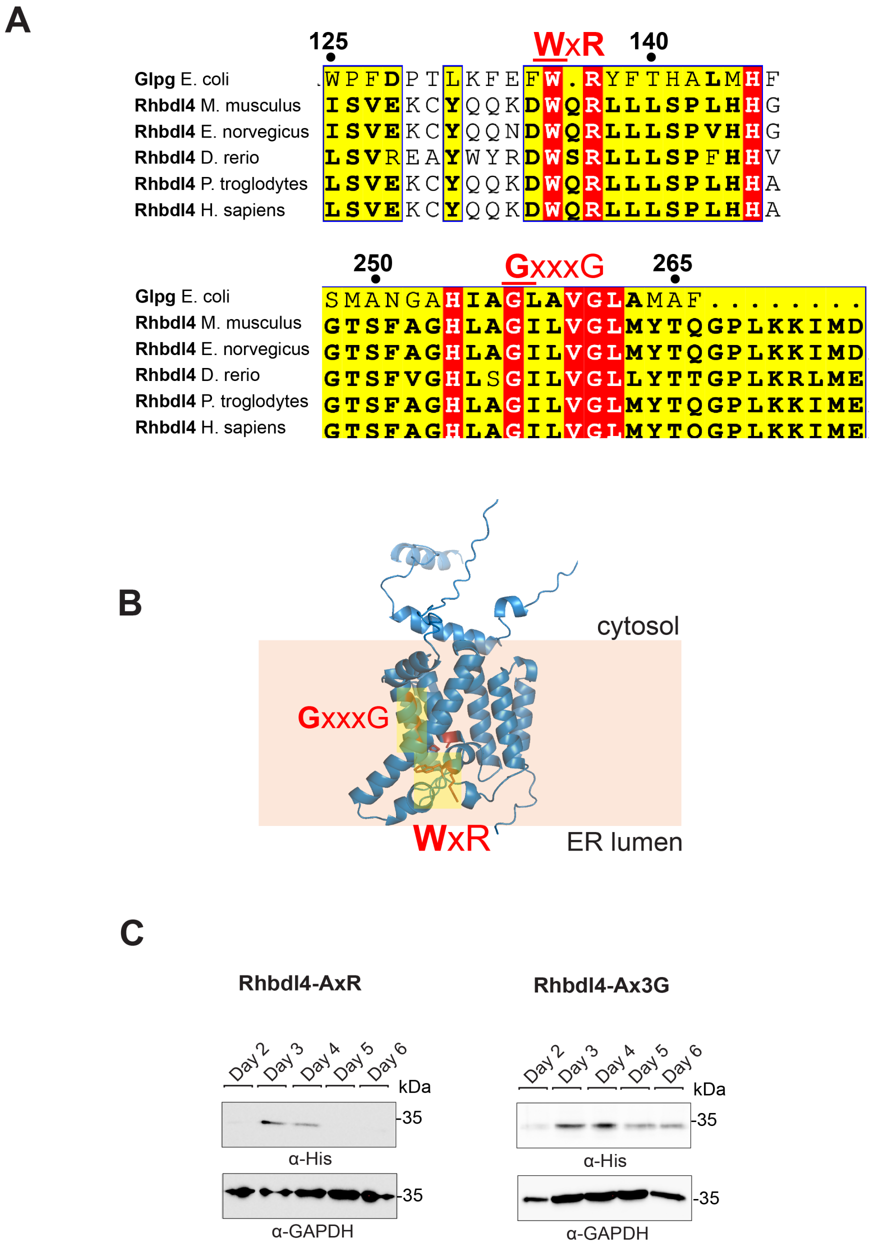

Supplemental Figure 3. (A) Conservation of RHBDL4. Alignment of RHBDL4 (*H. sapiens, M. musculus, E. norvegicus, D. rerio, P. troglodytes*) with GlpG (*E. coli*). Identical residues are highlighted in red. **(B)** Alphafold model of RHBDL4. Positions of WxR and Gx$_3$G motif is highlighted in yellow. **(C)** Freestyle 293-F suspension cells were transfected with RHBDL4-AxR and RHBDL4-Ax3G and cells were grown for the indicated amount of days. 50 µg of lysate was subjected to immunoblotting for RHBDL4 with monoclonal α-His antibody and monoclonal α-GAPDH antibody.

bioRxiv preprint doi: https://doi.org/10.1101/2024.10.13.618094; this version posted October 13, 2024. The copyright holder for this preprint (which was not certified by peer review) is the author/funder, who has granted bioRxiv a license to display the preprint in perpetuity. It is made available under aCC-BY 4.0 International license.

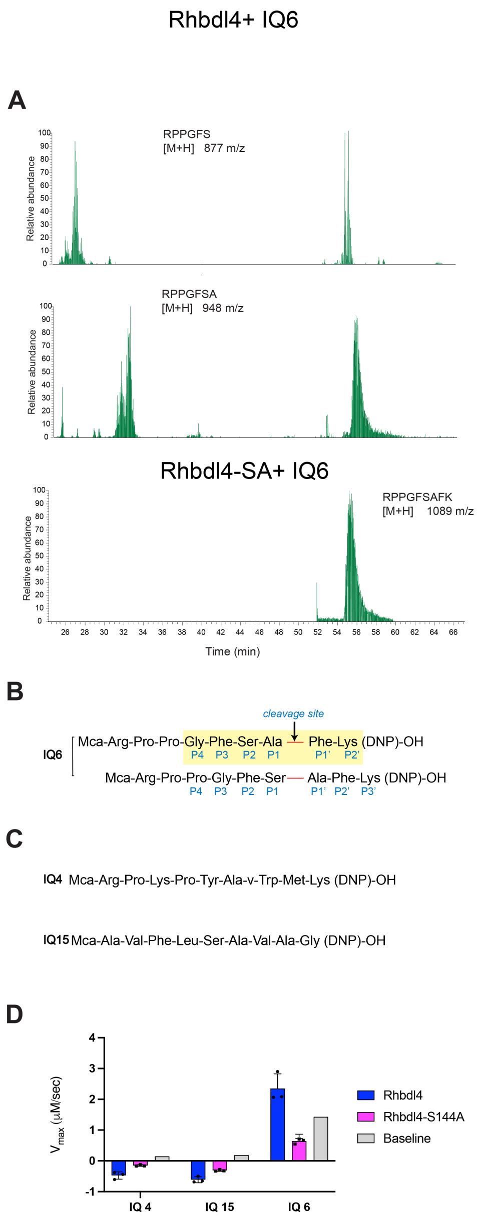

Supplemental Figure 4. (A) Wild-type RHBDL4 and inactive RHBDL4-S144A were incubated with 10 µM of the IQ6 substrate Mca-Arg-Pro-Pro-Gly-Phe-Ser-Ala-Phe-Lys (DNP)-OH for 4 h at room temperature, and the reaction was quenched with 8 M GuHCl, desalted, eluted, and analyzed via tandem mass spectrometry (MS/MS). **(B)** Depiction of IQ6 cleavage sites as determined from tandem mass spectrometry (MS/MS). **(C)** Peptide sequence of GlpG-derived and PARL-derived fluorescent substrates IQ4 and IQ15, respectively. **(D)** Activity profiles of the wild-type RHBDL4 and mutant RHBDL4-S144A, as measured by cleavage of the fluorescent substrates IQ4 and IQ15. Assays was performed in triplicate, and data points are represented by the average ± standard deviation

bioRxiv preprint doi: https://doi.org/10.1101/2024.10.13.618094; this version posted October 13, 2024. The copyright holder for this preprint (which was not certified by peer review) is the author/funder, who has granted bioRxiv a license to display the preprint in perpetuity. It is made available under aCC-BY 4.0 International license.

A

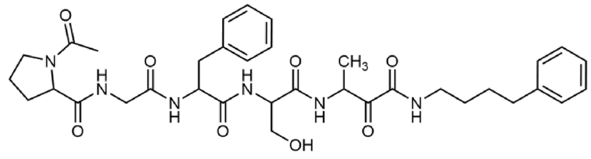

compound 2
IQ6-derived substrate (PGFSA)

B

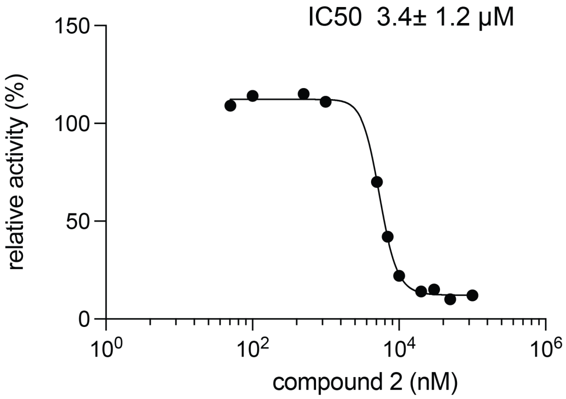

C

KSp207	
K_M(μM)	3.09±1
K_{cat}(hr^{-1})	11.05±0.8
K_{cat}/K_M($μM^{-1}hr^{-1}$)	3.54±.7

Supplemental Figure 5. (A) Schematic representation of the peptidyl- α-ketoamide inhibitor, compound **2. (B)** Representative inhibition curve derived from measuring rates of RHBDL4 proteolysis of the fluorescent substrate IQ6 with increasing concentrations of compound **2**. **(C)** Catalytic parameters of KSp207 substrate cleavage by RHBDL4.

bioRxiv preprint doi: https://doi.org/10.1101/2024.10.13.618094; this version posted October 13, 2024. The copyright holder for this preprint (which was not certified by peer review) is the author/funder, who has granted bioRxiv a license to display the preprint in perpetuity. It is made available under aCC-BY 4.0 International license.

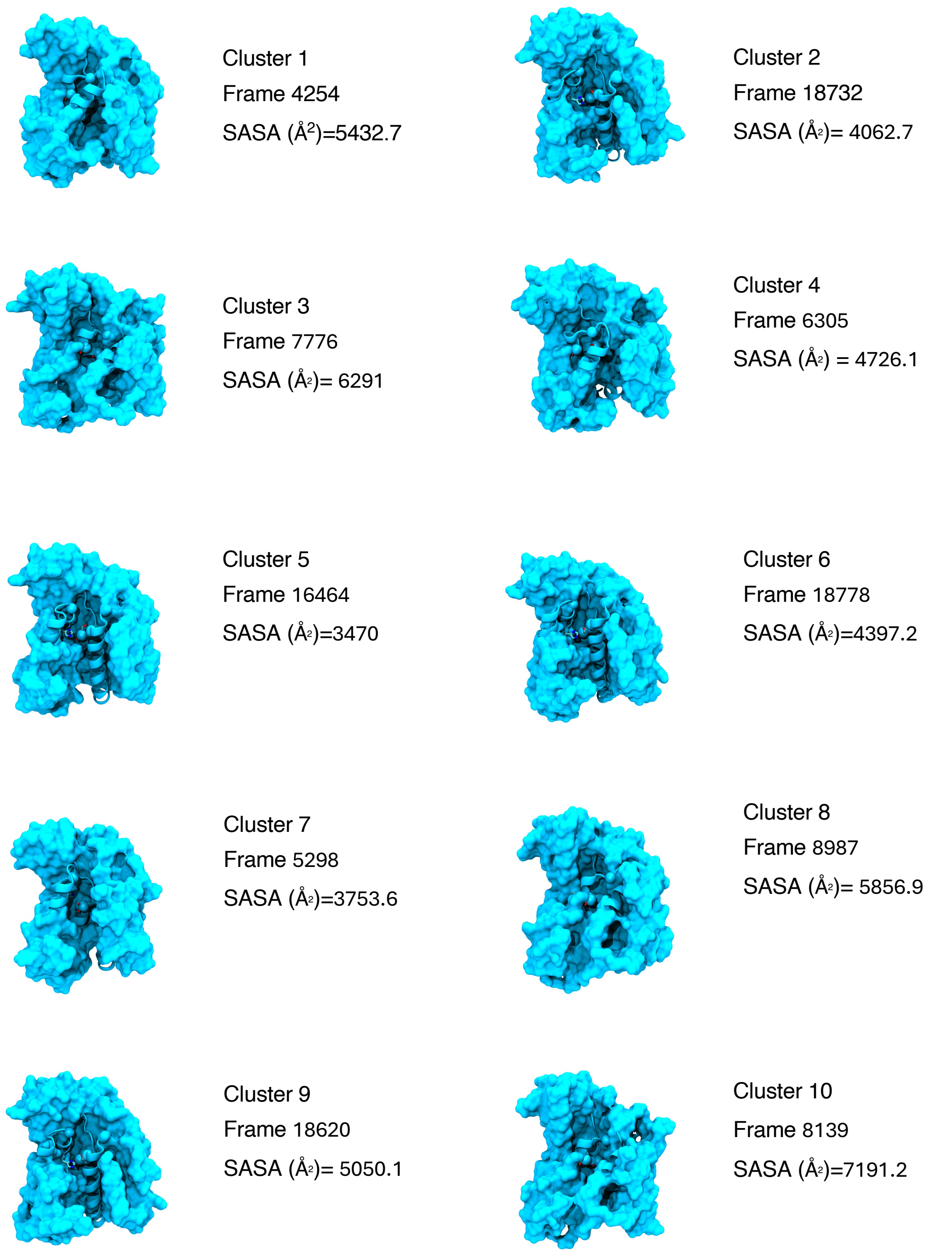

Supplemental Figure 6. RHBDL4 (apo) most populated clusters from all-atom MD simulations. Structures were clustered based from the solvent-accessibility-surface-areas (SASA) values of residues within 10 Angstrom of HIS144 and SER195. Each cluster was represented with the frames that has max SASA values.

bioRxiv preprint doi: https://doi.org/10.1101/2024.10.13.618094; this version posted October 13, 2024. The copyright holder for this preprint (which was not certified by peer review) is the author/funder, who has granted bioRxiv a license to display the preprint in perpetuity. It is made available under aCC-BY 4.0 International license.

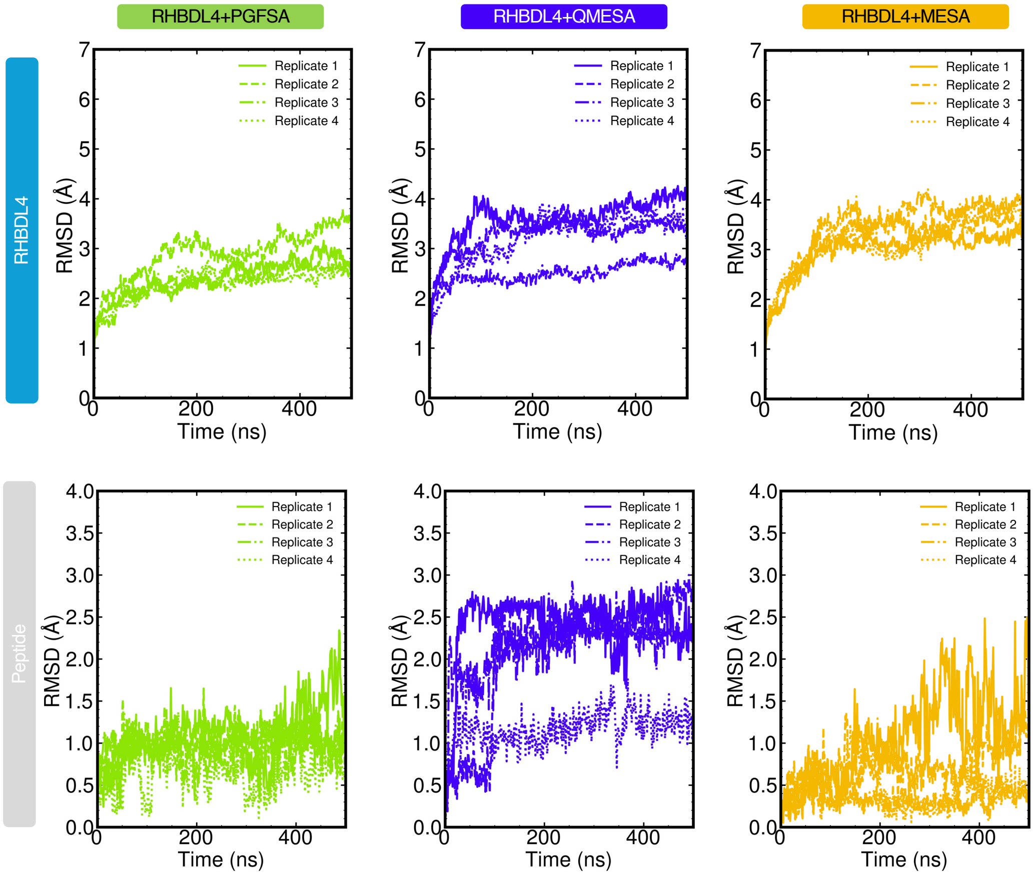

Supplemental Figure 7. RHBDL4 and peptide Ca Root-mean-square deviation from the initial frame of molecular dynamics simulations. Trajectories were aligned. Four replicates for each system were analyzed.

bioRxiv preprint doi: https://doi.org/10.1101/2024.10.13.618094; this version posted October 13, 2024. The copyright holder for this preprint (which was not certified by peer review) is the author/funder, who has granted bioRxiv a license to display the preprint in perpetuity. It is made available under aCC-BY 4.0 International license.

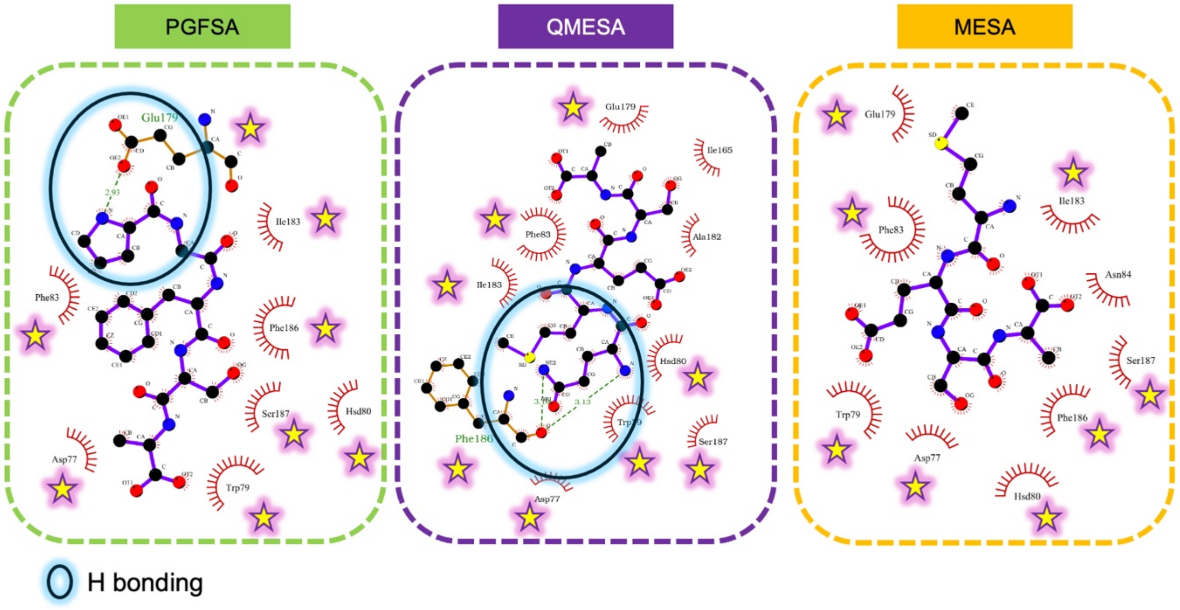

Supplemental Figure 8. 2D representation of RHBDL4 close interacting residues with the peptide side chains. All residues that were shared by the three substrate-derived peptides were marked with yellow stars. Ligplot+ was used to render this figure.

bioRxiv preprint doi: https://doi.org/10.1101/2024.10.13.618094; this version posted October 13, 2024. The copyright holder for this preprint (which was not certified by peer review) is the author/funder, who has granted bioRxiv a license to display the preprint in perpetuity. It is made available under aCC-BY 4.0 International license.

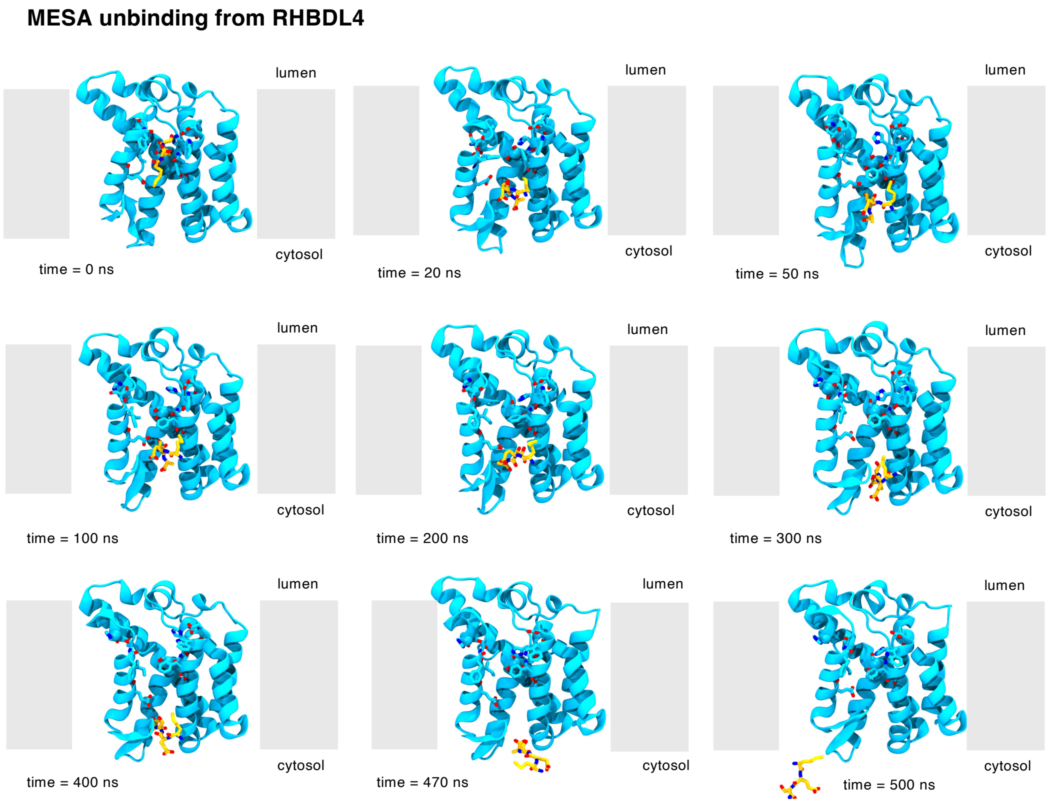

Supplemental Figure 9. Time evolution snapshot of MESA interaction with the RHBDL4 active site.

bioRxiv preprint doi: https://doi.org/10.1101/2024.10.13.618094; this version posted October 13, 2024. The copyright holder for this preprint (which was not certified by peer review) is the author/funder, who has granted bioRxiv a license to display the preprint in perpetuity. It is made available under aCC-BY 4.0 International license.

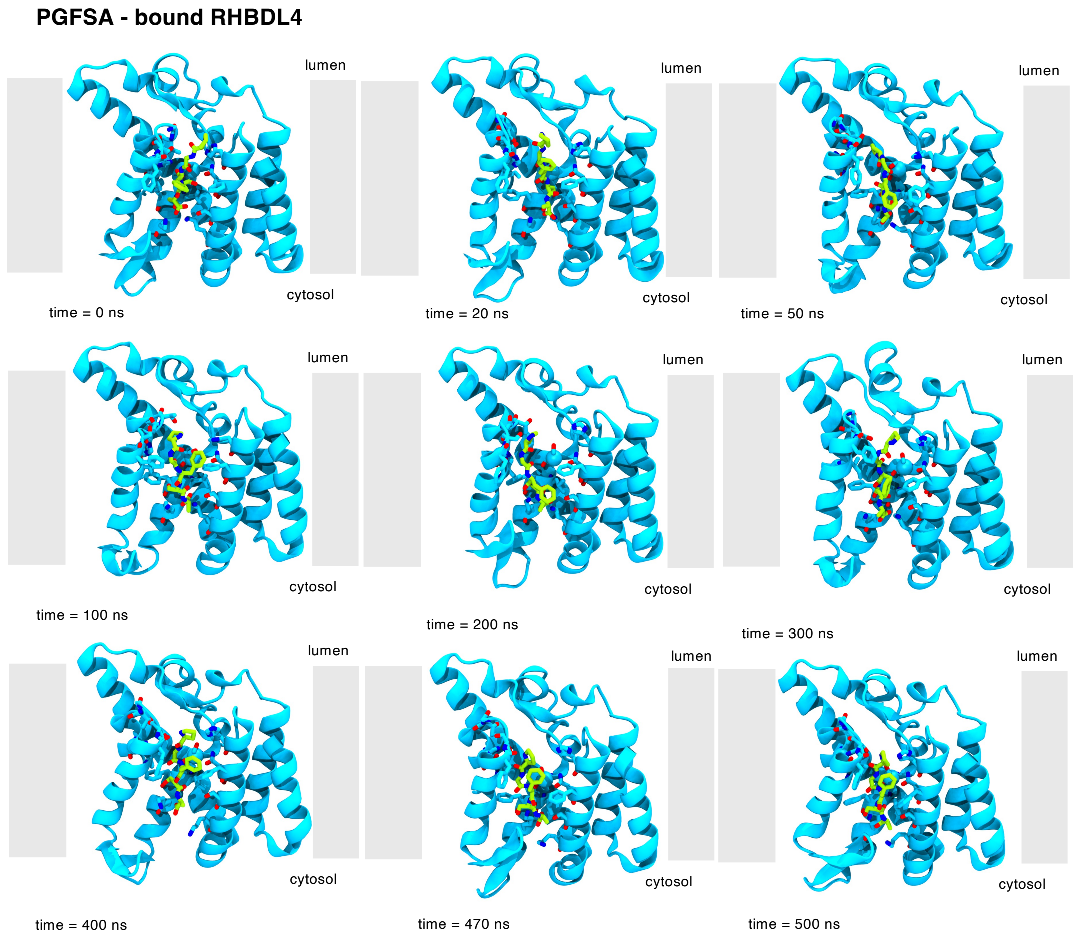

Supplemental Figure 10. Time evolution snapshot of PGFSA interaction with the RHBDL4 active site.

bioRxiv preprint doi: https://doi.org/10.1101/2024.10.13.618094; this version posted October 13, 2024. The copyright holder for this preprint (which was not certified by peer review) is the author/funder, who has granted bioRxiv a license to display the preprint in perpetuity. It is made available under aCC-BY 4.0 International license.

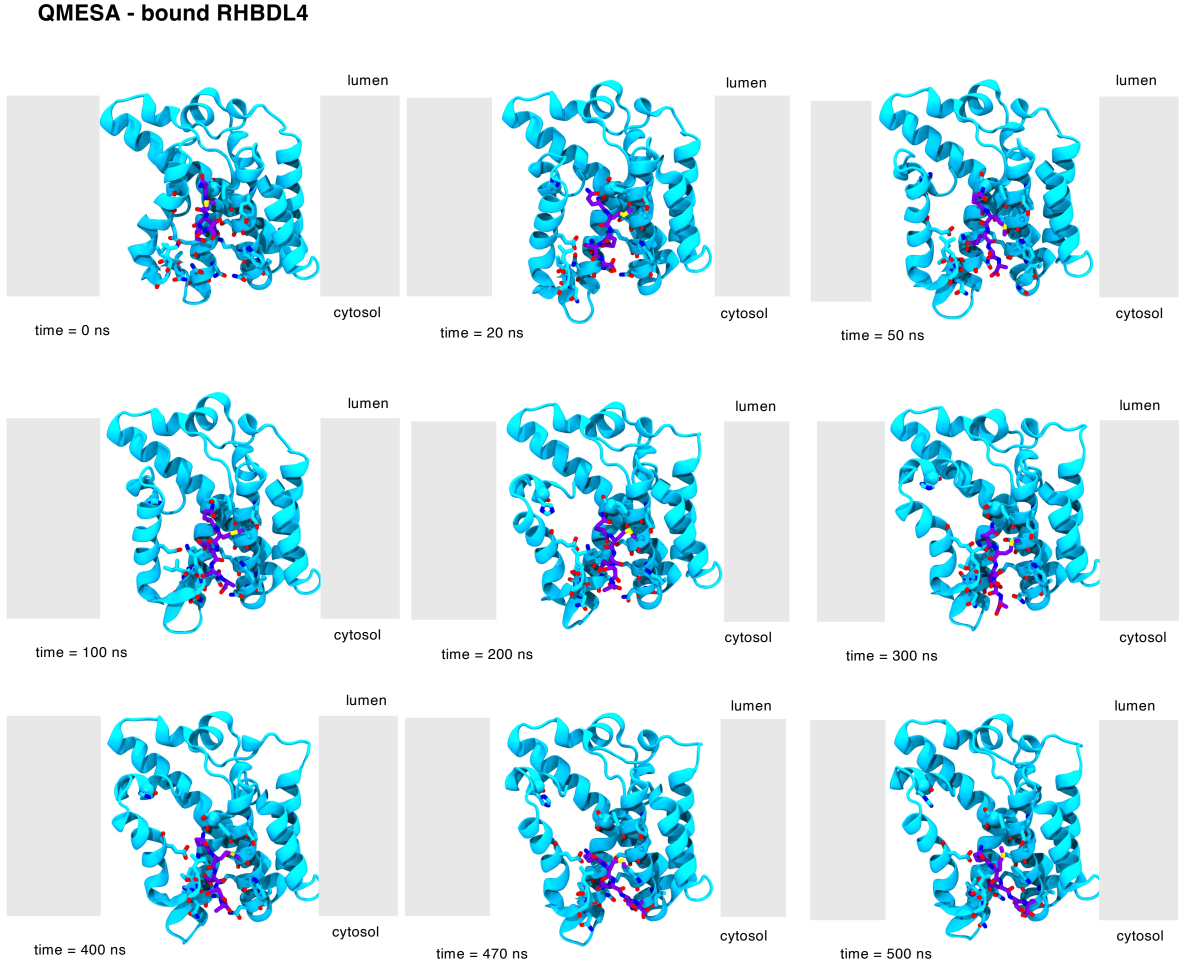

Supplemental Figure 11. Time evolution snapshot of QMESA interaction with the RHBDL4 active site.

bioRxiv preprint doi: https://doi.org/10.1101/2024.10.13.618094; this version posted October 13, 2024. The copyright holder for this preprint (which was not certified by peer review) is the author/funder, who has granted bioRxiv a license to display the preprint in perpetuity. It is made available under aCC-BY 4.0 International license.

Supplemental Table 1: AutoDock Vina ensemble molecular docking (n=4)

	**PGFSA (IQ6)** mca-RPPGFSAFK(dnp)	**QMESA (Ksp207)** Ac-QMESA-4mc	**MESA**
**Binding affinity** (kcal×mol^{-1})*	-6.8 ± 0.5	-5.2 ± 0.3	-5.2 ± 0.1

bioRxiv preprint doi: https://doi.org/10.1101/2024.10.13.618094; this version posted October 13, 2024. The copyright holder for this preprint (which was not certified by peer review) is the author/funder, who has granted bioRxiv a license to display the preprint in perpetuity. It is made available under aCC-BY 4.0 International license.

Supplemental Table 2. Plasmids used in this study

**Plasmid #**	**Backbone & Gene**
pSN182	pcDNA3.1 HisA RHBDL4
pSN183	pcDNA3.1 HisA RHBDL4-S144A
pSN294	pcDNA3.1 HisA RHBDL4-AxR
pSN295	pcDNA3.1 HisA RHBDL4-Ax3R
